# Dual-energy computed tomography for improved visualization of internal jugular chain neck lymph node metastasis and nodal necrosis in head and neck squamous cell carcinoma

**DOI:** 10.1007/s11604-023-01460-9

**Published:** 2023-06-22

**Authors:** Akira Baba, Ryo Kurokawa, Mariko Kurokawa, Roberto Rivera-de Choudens, Ashok Srinivasan

**Affiliations:** 1 Division of Neuroradiology, Department of Radiology, University of Michigan, 1500 E. Medical Center Dr., Ann Arbor, MI 48109 USA; 2 Department of Radiology, The Jikei University School of Medicine, 3-25-8 Nishi-Shimbashi, Minato-ku, Tokyo, 105-8461 Japan; 3 Department of Radiology, The University of Tokyo, 7-3-1 Hongo, Bunkyo-ku, Tokyo, 113-8655 Japan

**Keywords:** Dual-energy computed tomography, Head and neck cancer, Neck lymph node metastasis, Nodal necrosis

## Abstract

**Purpose:**

To evaluate and compare the utility of 40-keV virtual monochromatic imaging (VMI) reconstructed from dual-energy computed tomography (DECT) in the assessment of neck lymph node metastasis with 70-keV VMI, which is reportedly equivalent to conventional 120-kVp single-energy computed tomography.

**Materials and methods:**

Patients with head and neck squamous cell carcinoma who had neck lymph node metastasis in contact with the sternocleidomastoid muscle (SCM) and underwent contrast-enhanced DECT were included. In 40- and 70-keV VMI, contrast differences and contrast noise ratio (CNR) between the solid component of neck lymph node metastasis (SC) and the SCM and between SC and nodal necrosis (NN) were calculated. Two board-certified radiologists independently and qualitatively evaluated the boundary discrimination between SC and SCM and the diagnostic certainty of NN.

**Results:**

We evaluated 45 neck lymph node metastases. The contrast difference between SC and SCM and SC and NN were significantly higher at 40-keV VMI than at 70-keV VMI (*p* < 0.001). The CNR between SC and SCM was significantly higher at 40-keV VMI than at 70-keV VMI (*p* < 0.001). Scoring of the boundary discrimination between SC and SCM as well as the diagnostic certainty of NN at 40-keV VMI was significantly higher than that at 70-keV VMI (*p* < 0.001). The inter-rater agreements for these scores were higher at 40-keV VMI than at 70-keV VMI.

**Conclusion:**

Additional employing 40-keV VMI in routine clinical practice may be useful in the diagnosis of head and neck lymph node metastases and nodal necrosis.

## Introduction

Pretreatment evaluation of neck lymph node metastasis in head and neck cancer plays an important clinical role in appropriate management as it influences treatment planning and prognosis [1, 2]. Conventional 120-kVp single-energy computed tomography (SECT) often shows that neck lymph node metastases of head and neck cancers approximate the computed tomography (CT) density of muscle tissue [3]. Therefore, identification and detection of level II–IV lymph node metastases, belonging to the internal jugular chain in contact with the sternocleidomastoid muscle and constituting less surrounding fatty tissue than other neck lymph nodes, can be difficult due to the low contrast of their boundaries. Nodal necrosis, a key imaging feature of neck lymph node metastasis from head and neck cancer on contrast-enhanced CT or magnetic resonance imaging (MRI), is highly suggestive of neck lymph node metastasis [4]; thus, identifying this finding has clinical significance. Previous studies have demonstrated that low-keV virtual monochromatic imaging (VMI) reconstructed from dual-energy computed tomography (DECT) improves head and neck tumor visualization and soft tissue boundary discrimination by increasing tumor density and soft tissue contrast [5–9]. We hypothesized that this technique could improve the visualization of neck lymph node metastasis and nodal necrosis of head and neck cancer by increasing the contrast enhancement component of lymph node metastasis. This study aimed to evaluate the utility of 40-keV VMI reconstructed from DECT in the evaluation of neck lymph node metastases and nodal necrosis by comparing it with 70-keV VMI, which is considered equivalent to conventional CT with 120-kVp SECT [5, 6, 10].

## Materials and methods

This retrospective study obtained our institutional review board permission and the need for written informed consent was waived due to the retrospective nature of this study. All procedures were carried out in accordance with Helsinki Declaration.

### Patients

**Fig. 1:**
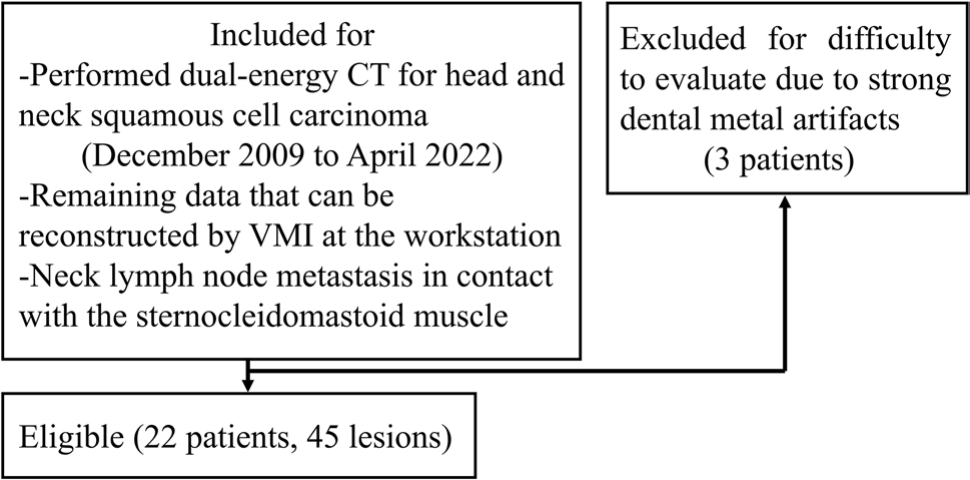
Flow chart of patient selection

The inclusion criteria of the study were: (1) patients with head and neck squamous cell carcinoma who underwent pretreatment contrast-enhanced DECT between December 2009 and April 2022, (2) had data available for retrospective VMI reconstruction, and (3) had neck lymph node metastases in contact with the sternocleidomastoid muscle (Fig. 1). Neck node metastasis was confirmed by either observing pathology or by positive ^18^F-fluorodeoxyglucose positron emission tomography (^18^F-FDG PET)/CT finding and imaging follow-up. Three patients, whose imaging of the neck was difficult to evaluate due to the strong presence of dental metal artifacts, were excluded. Positive CT findings of nodal necrosis (focal area of low attenuation with or without a surrounding rim of enhancement) [4] were judged by a board-certified radiologist with 13 years of experience in head and neck radiology who reviewed the 40- and 70-keV VMI of each lymph node metastasis.

### CT acquisition

CT examinations were performed using a 64-channel multidetector CT scanner (Discovery CT750 HD [*n* = 16], and Revolution Frontier [*n* = 4]; GE Healthcare, Chicago, IL, USA) and a 128-channel multidetector CT scanner (Revolution CT; GE Healthcare) (*n* = 2). All patients were scanned in the supine position from mid-orbit to the medial clavicles. The standard settings parameters included 1.25-mm slice thickness, 1.25-mm interval, 0.969:1 pitch, 335–640 mA, dual-energy of 80 and 140 kVp, 0.6–0.8-s rotation time, large-body scanning field of view (FOV), and 20 to 24-cm display FOV. Contrast was administered as a split bolus technique with an initial administration of 50 mL of iodinated contrast material (Isovue 300 [iopamidol], Bracco Diagnostics) at 2 mL/s. Following a 120-s delay, a second bolus of 75 mL of the same contrast material was administered at 2 mL/s (this technique was used as it optimizes contrast enhancement within neck lesions and, simultaneously, allows adequate contrast in vessels [8]), and the contrast-enhanced CT images were obtained 180 s after administering the first contrast. Post-processing using previously obtained spectral data on AW Server workstation, v3.2 Ext 4.0 (GE Medical Systems, Milwaukee, WI, USA) was performed by a board-certified radiologist with 13 years of experience in head and neck radiology. The 40- and 70-keV VMI were reconstructed for each scan, and they were rendered with the same window width and level.

### Image analysis

Two board-certified radiologists with 9 and 13 years of experience, respectively, in head and neck radiology, consented and placed three regions of interest (ROIs) in the solid component of neck lymph node metastasis, one ROI in the sternocleidomastoid muscle, and one ROI in the area of lowest CT density of nodal necrosis on 1.25-mm slice thickness CT. The average ROI size was 8 (range 2.6–14.2) mm^2^. ROIs were placed in the same area of 40- and 70-keV VMI using the workstation’s default settings, mainly referring to the 40-keV VMI, and one Hounsfield unit (HU) was calculated from each ROI. The values of the three ROIs in the solid component were averaged. The contrast difference between the solid component of neck lymph node metastasis (SC) and the sternocleidomastoid muscle (SCM) was calculated as SC minus SCM and the contrast difference between low density area inside the nodal necrosis (NN) and the SC located at the margins of the neck lymph node was calculated as SC minus NN. Contrast-to-noise ratio (CNR) between SC and SCM was calculated using the following formula: CNR = (SC − SCM)/square root [variance (SC) + variance (SCM)]. CNR between SC and NN was calculated using the following formula: CNR = (SC − NN)/square root [variance (SC) + variance (NN)] [11]. Two board-certified radiologists, with 9 and 6 years of experience, respectively, in head and neck radiology, independently evaluated each of the 40- and 70-keV VMI qualitatively on the boundary distinction between neck lymph node metastases and the sternocleidomastoid muscle as well as the diagnostic certainty of nodal necrosis in neck lymph node metastases. The level and width of the window were fixed at 50 and 350, respectively. The boundary discrimination between neck lymph node metastasis and the sternocleidomastoid muscle was defined as follows: 1 point, the boundary is unclear and cannot be determined; 2 points, the boundary is unclear but can be determined slightly; 3 points, the boundary is clearly identified. Diagnostic certainty of nodal necrosis was defined as follows: 1 point, equivocal; 2 points, probably; 3 points, definite.

### Statistical analysis

The decision to use parametric or nonparametric tests was made after evaluating the data distribution using the Shapiro–Wilk test. Contrast differences, CNR, and diagnostic certainty were compared between 40- and 70-keV VMI using the Wilcoxon signed-rank test. A *p* value < 0.05 was considered to indicate statistical significance. For qualitative evaluation of the boundary discrimination between SC and the SCM as well as the diagnostic certainty of NN, inter-examiner agreements in the evaluation were assessed using kappa analyses. Using standard criteria [12], inter-observer agreement was classified as poor (*k*, < 0.20), fair (*k*, 0.21–0.40), moderate (*k*, 0.41–0.60), good (*k*, 0.61–0.80), or very good (*k*, 0.81–1.00). The Fisher’s exact test was used to compare the relationship between the presence of pathologic extranodal extensions (ENEs) and the score between 0 and 1–2 in 70-keV VMI, as well as the score between 1 and 2 in 40-keV VMI. All statistical analyses were performed using R version 3.6.1 software (R Foundation for Statistical Computing, Vienna, Austria).

## Results

We evaluated 22 patients with head and neck cancer (male, *n* = 16; female, *n* = 6; average age ± standard deviation, 59.7 ± 8.6 years). Of 22 patients, the subsites of head and neck cancer included the larynx (*n* = 11), oropharynx (*n* = 8), hypopharynx (*n* = 2), and oral cavity (*n* = 1). The pathological T-staging categories ranged from T1 to T4a (T1, one patient; T2, five patients; T3, seven patients; T4, nine patients). Treatment included chemoradiotherapy in 14 patients, surgery combined with chemoradiotherapy in 7 patients, and surgery alone in 1 patient. A total of 45 neck lymph node metastases from 22 patients were included in the evaluation. They included 26 level II lesions and 19 level III lesions. The diagnosis of neck lymph node metastasis was based on positive pretreatment ^18^F-FDG PET/CT and imaging follow-up findings in 28 lesions and pathology in 17 lesions. Pathological ENE were identified in 11 of 17 lesions. The median size of the lymph nodes on CT was 18.9 (range, 12.0–46.9) mm. A total of 31 lymph node metastases with nodal necrosis CT findings were included in the evaluation.

**Fig. 2:**
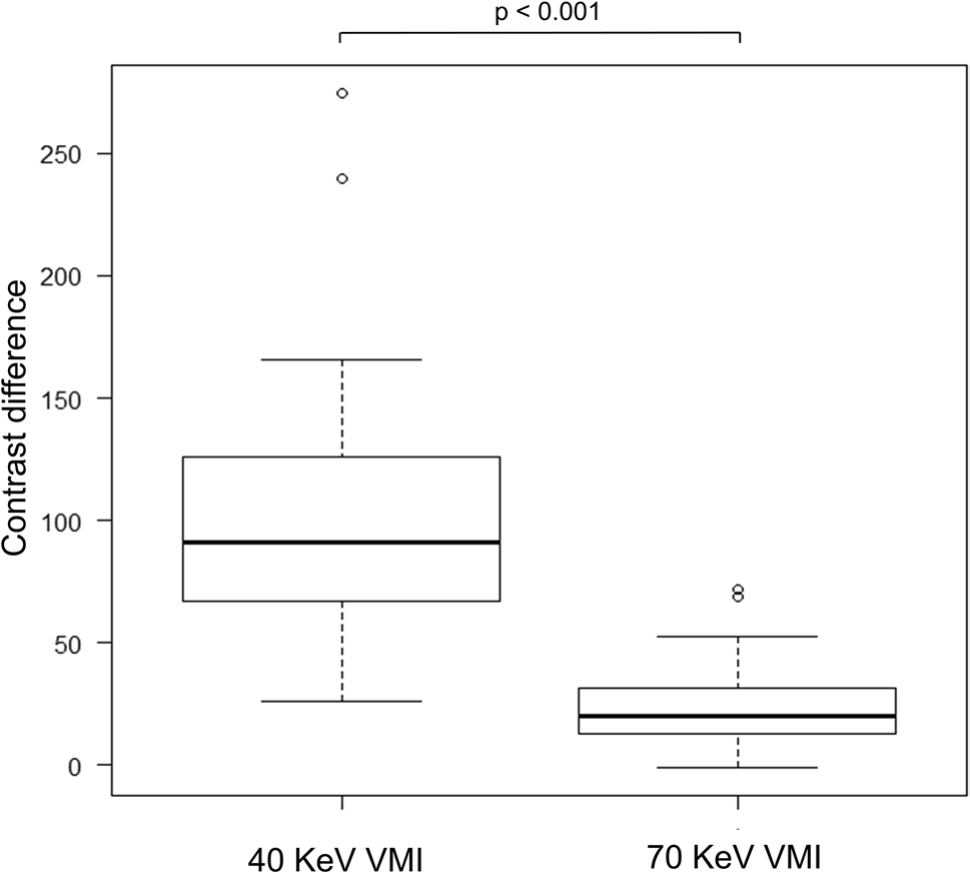
Box-and-whisker plots of the contrast difference between the solid component of neck lymph node metastasis and the sternocleidomastoid muscle in 40- and 70-keV virtual monochromatic imaging

**Fig. 3:**
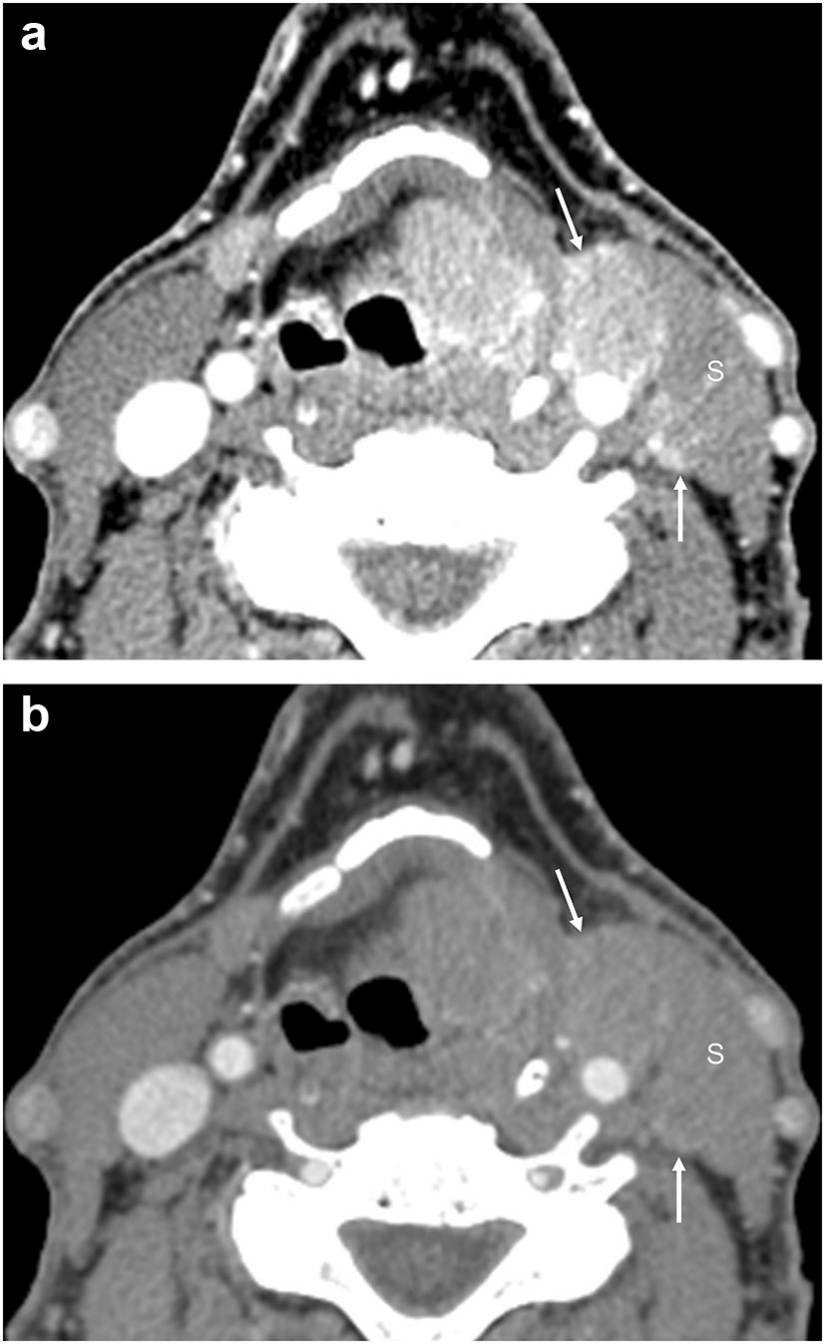
Virtual monochromatic imaging (VMI) of a neck lymph node metastasis. A 47-year-old male with supraglottic cancer. The 40-keV VMI (**a**) shows lymph node enlargement (arrows) at left level II, with a high contrast between the left level II lesions (arrows) and the sternocleidomastoid muscle (marked by S), and the boundary of the lesion is clearly defined. The 70-keV VMI (**b**) shows that the densities of the lesions and the left sternocleidomastoid muscle (marked by S) are almost equal, and the boundary is unclear

**Fig. 4:**
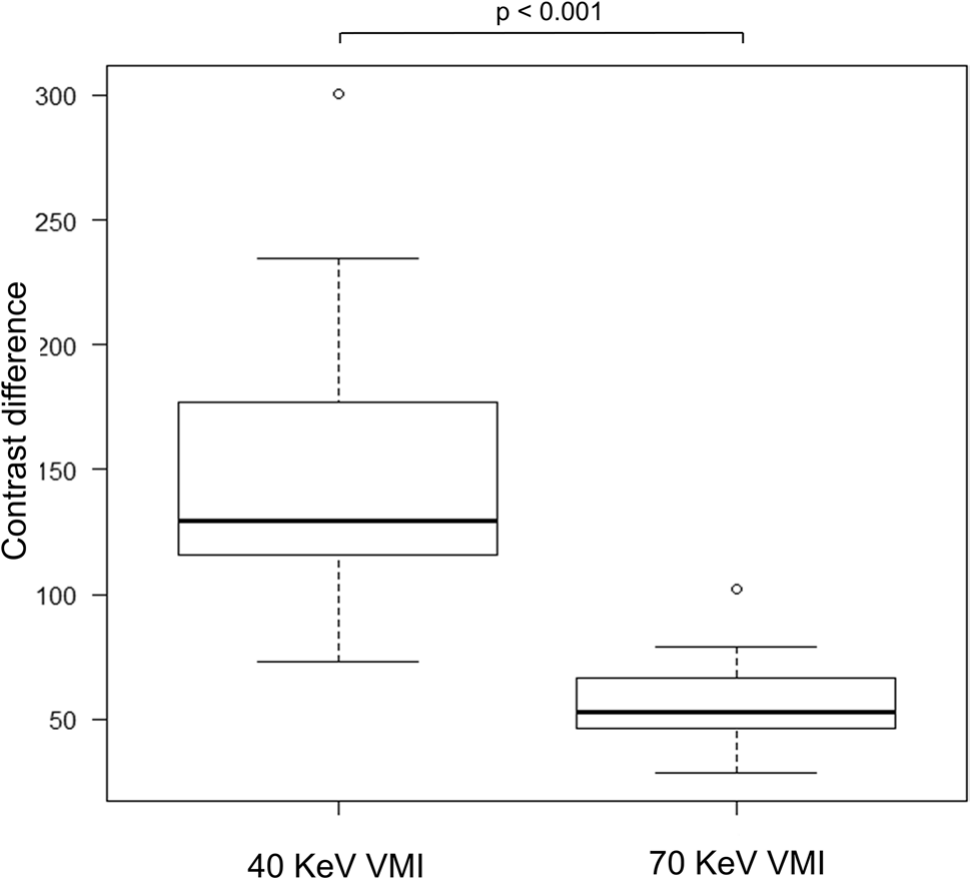
Box-and-whisker plots of the contrast difference between the nodal necrosis and the solid component of neck lymph node metastasis in 40- and 70-keV virtual monochromatic imaging

**Fig. 5:**
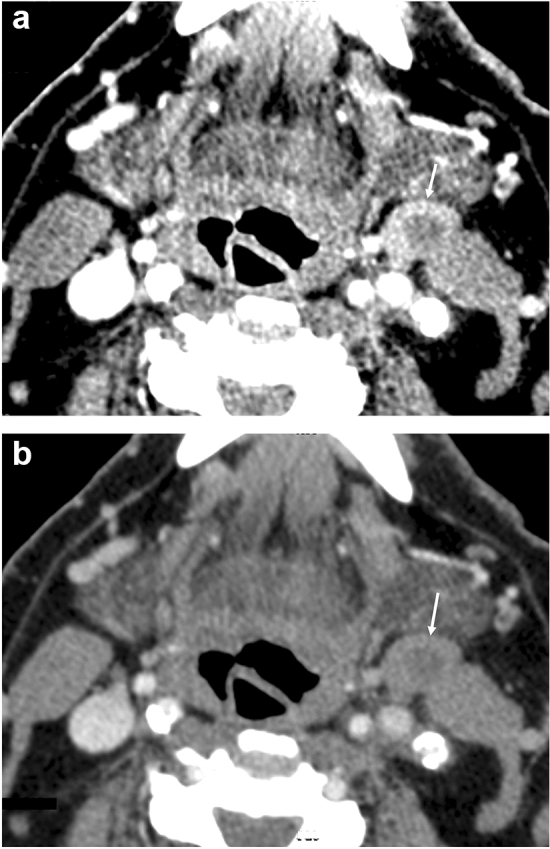
Virtual monochromatic imaging (VMI) of a nodal necrosis of neck lymph node metastasis. A 70-year-old male with supraglottic cancer. The 40-keV VMI (**a**) shows enlarged lymph node (arrow) at the left level II with a high contrast between the hypodense internal (arrow) and the solid component along the margins of the left level II lesion (arrow), and nodal necrosis can be judged definitively. The 70-keV VMI (**b**) shows the lesion with a slightly hypodense internal component, which is not definitive as a nodal necrosis

Th﻿e median HU values, along with their range, and contrast difference for each structure, are shown in Table 1. The difference between SC and SCM at 40-keV VMI was significantly higher than that at 70-keV VMI (*p* < 0.001) (Fig. 2), and a representative case is presented in Fig. 3. The difference between SC and NN at 40-keV VMI was significantly higher than that at 70-keV VMI (*p* < 0.001) (Fig. 4), and a representative case is presented in Fig. 5.

**Table 1:** Hounsfield unit values and contrast difference and CNR

	70-keV VMI	40-keV VMI
SC	82.7 (60.5 to 135.6)	192.0 (131.2 to 372.9)
SCM	63.0 (49.3 to 72.7)	97.6 (64.8 to 104)
NN	26.6 (11.2 to 50.2)	43.7 (− 14.5 to 128.1)
Difference b/w SC and SCM	19.9 (− 1.16 to 72.0)	90.8 (274.9 to 26.0)
Difference b/w SC and NN	52.5 (28.3 to 102.3)	129.6 (73.0 to 300.8)
Noise b/w SC and SCM	12.1 (3.9 to 25.2)	31.2 (9.5 to 56.7)
Noise b/w SC and NN	12.1 (5.2 to 25.6)	31.1 (10.7 to 55.8)
CNR b/w SC and SCM	1.8 (− 0.1 to 4.3)	3.3 (0.9 to 6.9)
CNR b/w SC and NN	4.4 (2.4 to 10.1)	4.8(2.4 to 11.0)

Values are presented as median (range)

*CNR* contrast noise ratio, *VMI* virtual monochromatic imaging, *SC* solid component of neck lymph node metastasis, *SCM* sternocleidomastoid muscle, *NN* nodal necrosis, *b/w* between

**Fig. 6:**
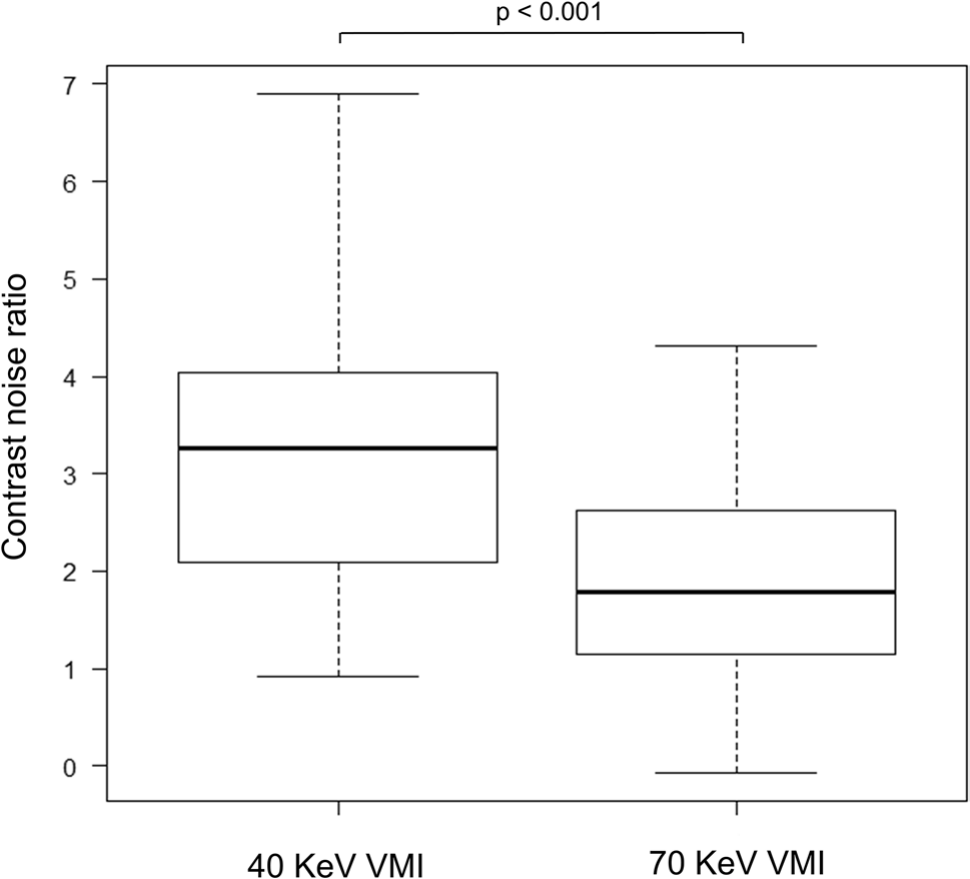
Box-and-whisker plots of the contrast noise ratio between the solid component of neck lymph node metastasis and the sternocleidomastoid muscle in 40- and 70-keV virtual monochromatic imaging

**Fig. 7:**
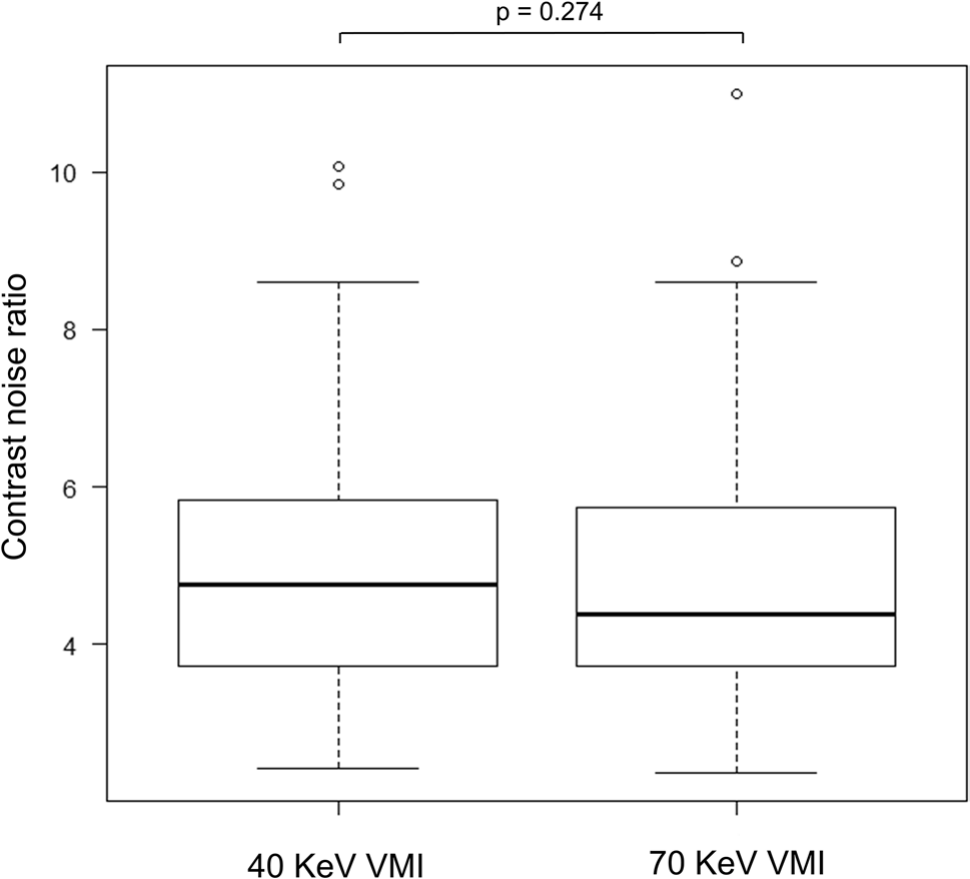
Box-and-whisker plots of the contrast noise ratio between the solid component of neck lymph node metastasis and the nodal necrosis in 40- and 70-keV virtual monochromatic imaging

The median noise, CNR between SC and SCM, and CNR between SC and NN for each structure are shown in Table 1. The CNR between SC and SCM at 40 keV VMI was significantly higher than at 70 keV VMI (*p* < 0.001) (Fig. 6). The CNR between SC and NN showed no significant difference between 40 keV VMI and 70 keV (*p* = 0.274) (Fig. 7).

The scoring of evaluators 1 and 2 on the boundary discrimination of neck lymph node metastases from the sternocleidomastoid muscle and the diagnostic certainty of nodal necrosis is shown in Tables 2 and 3. The scoring for the boundary discrimination between neck lymph node metastases and the sternocleidomastoid muscle at 40-keV VMI was significantly higher than that at 70-keV VMI for evaluators 1 and 2, respectively (*p* < 0.001). Scoring for diagnostic certainty of nodal necrosis at 40-keV VMI was significantly higher than that at 70-keV VMI for evaluators 1 and 2, respectively (*p* < 0.001).

**Table 2:** Scoring of evaluators 1 and 2 on the boundary discrimination of neck lymph node metastases from the sternocleidomastoid muscle (*n* = 45)

	Scoring	70-keV VMI (n)	40-keV VMI (n)
Evaluator 1	1	31	0
	2	12	13
	3	2	32
Evaluator 2	1	25	0
	2	15	16
	3	5	29

*VMI* virtual monochromatic imaging

**Table 3:** Scoring of evaluators 1 and 2 on the diagnostic certainty of nodal necrosis (*n* = 31)

	Scoring	70-keV VMI (*n*)	40-keV VMI (*n*)
Evaluator 1	1	2	0
	2	25	1
	3	4	30
Evaluator 2	1	1	0
	2	13	0
	3	17	31

*VMI* virtual monochromatic imaging

The agreement between evaluators 1 and 2 in scoring the boundary discrimination between neck lymph node metastasis and the sternocleidomastoid muscle was 0.53 (moderate) for 70-keV VMI and 0.65 (good) for 40-keV VMI, indicating an increase in agreement. The agreement between evaluators 1 and 2 in scoring the diagnostic certainty of nodal necrosis was 0.23 (fair) for 70-keV VMI and 0.95 (very good) for 40-keV VMI, indicating an increase in agreement. The presence of pathological ENEs was not significantly different from the results of score 0 or 1–2 in 70-keV VMI and score 1 or 2 in 40-keV VMI (*p* = 0.52–0.99).

## Discussion

The 40-keV VMI had significantly higher contrast difference between the SC and the SCM and between the SC and NN when compared to that of the 70-keV VMI. The CNR between SC and SCM at 40-keV VMI was significantly higher than that at 70-keV VMI. Compared with the 70-keV VMI scores for the boundary discrimination of neck lymph node metastases from the sternocleidomastoid muscle and the diagnostic certainty of nodal necrosis, those of the 40-keV VMI were significantly improved for each evaluator, as was the agreement rate among the evaluators.

DECT is a CT technique that allows the acquisition of images generated from two separate peak energy X-ray spectra. This technique enables the acquisition of data useful for material separation, as well as image reconstruction, such as iodine maps, virtual unenhanced images, and VMI [13, 14]. Several studies using DECT have demonstrated that low-keV VMI improves tumor visualization and soft tissue boundary discrimination by increasing soft tissue contrast in head and neck tumors [5–9]. One of these studies included neck lymph node metastases (*n* = 17) in addition to primary head and neck squamous cell carcinoma [5]. In contrast, our study included more neck lymph node metastases (*n* = 45) and had the advantage of focusing only on neck lymph node metastases. Moreover, we found that 40-keV VMI provided higher contrast difference and CNR between the SC and the SCM than 70-keV VMI, increased the score for boundary discrimination between the SCM and neck lymph node metastases among evaluators, and showed increased inter-rater agreement. Photons with energy near the k-edge of iodine (33 keV), especially low-energy photons (e.g., near 40 kVp), undergo photoelectric absorption by the iodine atom. Thus, low-kVp CT images demonstrate high HU for tissues containing iodine [15]. This may explain the support for the results of the current study. The results of our study suggest that 40-keV VMI may be useful in detecting and assessing the extent of neck lymph node metastases, especially in level II–IV lymph node metastases that may be adjacent to or in contact with the sternocleidomastoid muscle, by enhancing contrast difference and physical image quality with the surrounding lesion in routine clinical practice.

NN is an imaging finding on contrast-enhanced CT and MRI in neck lymph node metastases of head and neck cancers. It is a prognostic factor for ENE [16] and indicates a high probability of metastasis [4] and poor prognosis [17]. Therefore, the ability to detect this finding with better visualization and a high diagnostic certainty is of great clinical significance for appropriate diagnosis and treatment management. To date, there have been no studies on NN using DECT. Head and neck abscesses, which differ in pathology and clinical presentation but have relatively similar morphology on contrast-enhanced CT, have been reported to have the potential to improve evaluation utility and diagnostic certainty with VMI in DECT [18]. The results of this study show that 40-keV VMI has a higher contrast difference between the SC and the NN of neck node metastases than 70-keV VMI, an increased score on the diagnostic certainty of nodal necrosis in the evaluators, and an increased inter-rater agreement. This may be the result of the k-edge of iodine characteristics as well as the previously mentioned description [15]. Thus, 40-keV VMI might enhance the contrast of solid and necrotic tissue in metastatic lymph nodes and may be useful in detecting the finding in routine clinical practice.

Although the 40-keV VMI shows improvement of contrast and confidence in detecting neck node metastasis and nodal necrosis, there is the problem of stronger noise. The utility of noise reduction in iterative reconstruction or similar reconstruction methods has been reported [19]. Future studies are warranted to verify validity of the results of this study with the addition of noise reduction.

The results of the current study can be useful for qualitative evaluation in clinical practice. Studies of the diagnosis of lymph node metastasis using DECT quantitative parameters (electron density, effective atomic number in the non-contrast phase and iodine concentration [IC], normalized IC, slope of the energy spectrum curve, and dual-energy index) have been reported [20–23]. The comprehensive evaluation of these quantitative parameters, in combination with the qualitative results of the current study, may further improve the diagnostic performance of neck lymph node metastases in head and neck cancer. Further studies are warranted to validate this hypothesis.

This study has some limitations. First, this was a retrospective, single-center study with a small number of patients, that is, low number of pathologically diagnosed lesions and eligible lesions that were not pathologically proven but evaluated by PET/imaging follow-up. These factors reduce the generalizability of our data. Although latter validation method has been used in a previous study [24], the evidence for diagnosing metastasis based on positive PET uptake may be insufficient. Second, although the study indicated improved contrast difference and confidence in the detection of nodal necrosis, the CNR showed no significant differences; additionally, we could not prove whether they contributed to the diagnostic performance for the presence of lymph node metastases. Third, the gold standard for nodal necrosis is the evaluation of CT findings by a skilled head and neck radiologist. This method may cause selection bias. Last, since 40-keV VMI tends to be noisy, it might be difficult to distinguish micronecrosis from image noise or lymph follicles. The method of contrast injection in this study is not commonly used, and may have exerted an effect on the study result.

## Conclusion

Compared with 70-keV VMI, the 40-keV VMI reconstructed from DECT improved the contrast difference and confidence in detecting head and neck lymph node metastases and nodal necrosis. Additional employing 40-keV VMI in routine clinical practice may be useful in the diagnosis of head and neck lymph node metastases and nodal necrosis.
